# Evaluation of a Tannin Blend on Beef Cattle Performance and Health During the Receiving Period and Subsequent Grazing Period

**DOI:** 10.3390/vetsci12090833

**Published:** 2025-08-29

**Authors:** J. Daniel Rivera, Miriam A. Snider, Cody T. Shelton, R. Cyle Jones, Grayson Gourley, G. Doug Hufstedler, F. Henry Hilscher

**Affiliations:** 1 Southwest Research & Extension Center, Division of Agriculture, University of Arkansas System, Hope, AR 71801, USA; msnider@uada.edu (M.A.S.); ctshelto@uada.edu (C.T.S.); richardj@uada.edu (R.C.J.); ggourley@uada.edu (G.G.); 2 Silvateam USA, New York, NY 10022, USA; dhufstedler@silvateam.com; 3 Livestock Nutrition Center, Chickasha, OK 73018, USA; hhilscher@lnc-online.com

**Keywords:** beef cattle, bovine respiratory disease, grazing, tannin blend performance

## Abstract

One hundred thirty-three high-risk crossbred beef steers and bulls (178.2 ± 20.9 kg) were used to evaluate dietary inclusion of a tannin (BXA) blend pellet during a 63 d receiving period and a 126 d grazing period compared with no dietary treatment (CON). Data were analyzed using SAS 9.4 with pen as the experimental unit. During receiving, no differences were noted on average daily gain (ADG) on a deads-out (*p* > 0.25) or deads-in (*p* > 0.75) basis. There were tendencies for increased DMI associated with BXA from D0–21, D23–42, and D0–42 (*p* < 0.13). During the grazing period, cattle fed BXA had greater ADG from D0–56, and D0–84 (*p* ≤ 0.05), and tended to have greater ADG from D0–28, D0–112, and D0–126 (*p* ≤ 0.09). Total period ADG (deads-out) did not differ between treatments (*p* = 0.18) nor on a deads-in basis (*p* = 0.20). No differences were noted in the percentage of animals treated for bovine respiratory disease (*p* = 0.19), death loss (*p* = 0.62), or percentage of animals removed as chronically morbid/dead (*p* = 0.90). Results indicate that BXA did not improve performance or health during the receiving period. However, this additive had positive effects during the grazing period.

## Simple Summary

Traditionally antibiotics have been used as a method for improving animal health and well-being. However, greater scrutiny has been placed on the use of antibiotics within animal agriculture. Therefore, alternative methods to control bovine respiratory disease should be evaluated. This study evaluated the use of a tannin blend product on the health and performance of crossbred beef cattle during the receiving period, as well as the long-term effects of this product on grazing performance. Results indicate that feeding the tannin blend product to cattle at receiving did not improve health or performance. However, during the subsequent grazing period, the tannin blend increased animal performance.

## 1. Introduction

Bovine respiratory disease (BRD) continues to pose challenges to the beef cattle industry. It is estimated that 16.2% of all cattle placed in feedlots are affected by BRD [1]. However, this number does not consider cattle from other segments such as the cow–calf or stocker sectors. Most research has focused on the use of vaccines, antimicrobial therapies, probiotics or other management strategies to mitigate these losses [2]. There is greater scrutiny placed on the use of antibiotic use in beef cattle production due to potential concerns regarding antimicrobial resistance [3]. Therefore, alternatives to antibiotics have been explored. Some work has involved the use of antioxidants as methods to improve immune response, specifically vitamin E [4]. Vitamin E has been shown to potentially reduce morbidity from BRD [5] and has been effective in increasing cell-mediated antibody response [6], potentially through its antioxidative function. Stress has been indicated to have a negative effect on immune response and can lead to partial immunosuppression [7] due to excessive free radical production, which could lead to inflammation [8]. Antioxidants like vitamin E can help counter the negative effects of free radical production. Other compounds with antioxidant properties, such as polyphenols, have also shown to have similar effects on health responses. Polyphenols from grape leaves have demonstrated the ability to enhance humoral immunity in avians [9] and rabbits [10], and have some effect on the immune response of dairy cows [11]. However, some research has demonstrated that this antioxidant activity may be degraded in the rumen, thereby reducing their antioxidant activity. Engler et al. [12] were able to enhance immune response to vaccines using a rumen-protected polyphenol compound in dairy cows.

Tannins, rich in polyphenols, have long been regarded as an antinutritional factor in ruminant diets. However, they have also shown promise as a method to improve health and performance in ruminants when fed at low to medium concentrations. Tannins have been demonstrated to bind proteins in the rumen, thereby increasing the availability of amino acids for production [13]. In a review [14], it was noted that increasing amino acids to livestock can result in improved immune response. However, most of the work cited was in poultry and few studies were observed in ruminants. Tannins can affect the amount of amino acids that are absorbed by the small intestine in sheep [15]. In a meta-analysis it was determined that dietary tannin supplementation reduced ammonia N in the rumen, and improved ruminal propionate and butyrate [16]. In addition to potential growth responses, amino acids have been shown to possibly provide oxidative regulation during periods of oxidative stress in dairy cows [17].

In addition to the potential for increasing amino acid availability to the ruminant, tannins also may reduce methane (CH_4_) excretion by the ruminant [16] without any adverse effects. It has been reported that a combination of condensed tannins (CT) and hydrolyzed tannins (HT) were able to elicit a positive growth response in Holstein steers compared with either condensed or HT alone [18]. Similarly in meta-analyses, it was determined that tannins were able to reduce CH_4_ emissions and urinary N excretion, [16,19]. Despite these metabolic differences, no effect was detected regarding weight gain [16]. In contrast, when tannins are blended with bioflavonoids and essential oils, improvements in growth, feed efficiency, and health were improved in Charolais bulls [20]. In veal calves, a similar blend of tannins, bioflavonoids, and essential oils resulted in improved performance, and reduced relapses to BRD [21]. It has been demonstrated that, by modulating the rumen environment and reducing CH_4_ through the use of tannins, there can also be an improvement in immune response [21].

Therefore, the scope of this study was to determine the effects of feeding a proprietary blend of tannins, saponins and polyphenols (Silvateam, New York, NY, USA) to lightweight, newly received beef cattle on health, receiving period performance, and subsequent grazing performance.

## 2. Materials and Methods

### 2.1. Animal Care and Use

All procedures were conducted under the auspices of the University of Arkansas, Division of Agriculture Institutional Animal Care and Use Committee (Protocol Number 25075).

### 2.2. Receiving Period

One hundred thirty-three English crossbred steers and bulls (BW = 178.2 ± 20.9 kg) were purchased and shipped 919 km from an order buyer facility in Montgomery, AL to the University of Arkansas System, Division of Agriculture, Southwest Research & Extension Center Stocker Unit in Hope AR. Calves arrived at the Stocker Unit at approximately 2200 h, were unloaded, and provided access to hay and water. The following morning at 0700, cattle were brought to the working facilities and processed. Processing included (1) obtaining an individual body weight (BW), (2) applying a uniquely numbered eartag, and (3) vaccination against respiratory (Pyramid 5 + Presponse, Boehringer Ingelheim Animal Health, St. Joseph, MO, USA) and clostridial pathogens (Bovilis Covexin 8, Merck Animal Health, Rathway, NJ, USA). A fecal sample was collected for parasite analysis and each animal was treated with an injectable anthelmintic (Doramectin, Dectomax, Zoetis, Kalamazoo, MI, USA). Animals were classified as bull or steer based upon palpation. Cattle were then stratified by castrate status (bulls vs. steers) and sorted by BW. Body weight was used as a block and within block, cattle were assigned to pens with two pens per block with 11 or 12 steers per pen. Due to stratification, the percentage of intact males was equal across treatments (78% bulls). Pens within a block were randomly assigned to treatments using a random number generator. Following stratification and sorting, cattle were weighed again and assigned a second eartag corresponding to pen number. Intact males were surgically castrated and each steer was given a metaphalaxis of tilmicosin (Micotil, Elanco, Greenfield, IN, USA) and moved to their home pen. The time between the beginning of processing and final movement to their home pen was approximately 5 h.

### 2.3. Treatments

Receiving diets were fed to reach a target limited rate (2.5% of BW). Cattle received one of two experimental diets: (1) a control diet (CON) or (2) a similar diet supplemented with Silvateam BXA pellet (BXA; Table 1) Diets were similar in formulation, except that the BXA diet contained a tannin blend pellet designed to deliver 7 g of BX d/head and 1 g of ATX d/head (per manufacturer recommendations). SilvaFeed BX contains a blend of quebracho (CT) and chestnut (HT) tannins as well as mixed screenings selected for their saponin content. SilvaFeed ATX contains a blend of quebracho (CT) and chestnut (HT) tannins as well as mixed screenings selected for their polyphenol rich profile. Cattle were fed twice/d at 0800 and 1400 with a 50:50 split of the daily feed amount. Cattle were initially fed 50% of their total feed with feed offered gradually increased based upon how well they consumed the previously offered feed. Diets were sampled once weekly, compiled, and analyzed for dry matter (DM) content. Dried samples were then submitted to a commercial laboratory (Midwest Labs, Omaha, NE, USA) for nutrient analysis.

### 2.4. Animal Health

During the receiving period, cattle were observed daily for symptoms of BRD using the clinical illness score (Table 2). Once the 5-d post-treatment interval (PTI) on the metaphalaxis was reached, cattle scoring ≥ 2 on the score were removed from the pen and taken to the working facilities for examination. If rectal temperature (RT) was greater than 39.7 °C, the animal was considered morbid and treated with antibiotics. Cattle that did not have a RT of greater than 39.7 °C, but were scoring ≥ 3 or who had been pulled and evaluated 2 d in a row were also considered morbid and treated with antibiotic therapy. Antibiotic treatment and PTI were as follows: 1st treatment was tulathromycin (Draxxin, Zoetis Kalamazoo, MI, USA) with a 7-d PTI, 2nd treatment was florfenicol (Norfenicol, Norbrook Laboratories, Lenexa, KS, USA) with a 3-d PTI, and 3rd treatment was oxytetracyline, (Liquamycin LA-200, Zoetis, Kalamazoo, MI, USA) with a 3-d PTI. Cattle that continued to display symptoms after the 3rd treatment were considered chronically ill and removed from the study.

### 2.5. Animal Management During the Receiving Period

Cattle were maintained in a partially covered confined feeding area during the receiving period. Feedlot pens (7.31 m × 18.3 m) were in an open-sided barn with a 6.1 m linear concrete bunk positioned towards the center of the barn for feeding. Each pen had a self-filling water trough (75 L). Pens were composed of a soil surface with approximately 2.5 m of a concrete apron at the front of each pen.

Cattle were individually weighed on D21, D42 and D63 with an unscheduled weigh d on D14. Due to excessive morbidity, poor feed intake, and general poor performance, cattle were individually weighed and treated with an oral anthelmintic (fendbendazole, Safe-Guard, Merck Animal Health, Rathway, NJ, USA). As this was an unscheduled event, data were not used in the final statistical analysis.

Feed was designated to be fed at a limited rate (2.5% of BW, DM-basis) with feed intake targets changed after each scheduled weigh period to reflect the new target BW. Prior to weighing cattle, any refused feed was collected, weighed, and used to adjust dry matter intake (DMI). Total feed offered during each 21-d period was adjusted for DM (based upon weekly samples), adjusted for any refused feed, then divided by head d (number of animals × number of d). If an animal died or was removed from the study, the pen feeding target was adjusted by subtracting that animal’s previous weight. Average daily gain and feed efficiency (gain:feed; G:F) were calculated using a “deads-in” and “deads-out” value and reported both ways. Deads-in calculations left the dead animal in the total calculations, whereas deads-out removed the dead animal from all calculations. On D63 cattle were weighed and maintained in their pens for 3 d, at which time they were weighed again, and then moved to cool-season annual pastures.

Diet samples were collected within each weigh period, compiled, then submitted to a commercial laboratory for analysis via wet chemistry (Midwest Labs, Omaha, NE, USA). Laboratory results are presented in Table 3.

### 2.6. Grazing Period

Following the receiving period, the remaining cattle (91 head) were allocated to graze a blend of annual ryegrass (*Lolium multiflorum*) and cereal rye (*Secale cereale*). Briefly, pastures were planted with 28 kg/ha of annual ryegrass and 90 kg/ha of cereal rye using a no-till drill in mid-October. Cattle were housed in 1.6 ha paddocks and maintained in their same pen groups. There was 3.7 m of linear bunk space per pen group. In addition to grazing, cattle had access to soybean hulls fed at the rate of 0.75% of their BW (CON), or soybean hulls fed 0.75% of their BW with the added BXA pellet to provide 8 g of the tannin blend. Cattle were maintained on the same treatments they were assigned to during the receiving period. Soybean hulls were primarily used as a method to deliver BXA to cattle. However, to account for any potential effects of supplementation on cattle performance, CON animals were fed soybean hulls at the same rate without the BXA pellet. Samples of soybean hulls were collected and analyzed for DM and, once dried, were submitted to a commercial laboratory for nutrient analysis (Midwest Labs, Omaha, NE, USA). Overall nutritive value of soybean hulls was 10.8% crude protein (CP) and 68.1% total digestible nutrients (TDN; DM-basis). During the grazing period, if grass became limiting (forage biomass < 897 kg/ha) the amount of soybean hulls fed were increased. Additionally, during the grazing period, hay was offered during excessively cold periods of time (snow and freezing rain events). Subsequent weigh periods were on a pen-basis every 28 d for 112 d. Cattle were weighed individually two wk later on D126 upon termination of the study. The study was terminated on D126 due to inadequate forage conditions. Following each weigh period, forage biomass measures were collected every 28 d ± 2 d using a calibrated rising plate reader with samples collected for nutrient analysis (Midwest Lab, Omaha NE) and are reported in Table 4. Biomass was simply used to determine whether supplementation needed to be altered.

### 2.7. Statistical Analysis

Receiving performance data were analyzed using the MIXED procedure of SAS 9.4 with pen as the experimental unit and block as a random effect. Health responses (morbidity, mortality, and animals removed) were analyzed using the PROC GLIMMIX procedure of SAS. Distribution of morbidity across d was analyzed using PROC GLIMMIX with a binomial distribution. Grazing performance data were analyzed using PROC MIXED in SAS, using the same model as the receiving period. Significance was declared at *p* ≤ 0.05 and tendencies were declared at *p* ≤ 0.15.

## 3. Results

### 3.1. Receiving Period

Data from the receiving period are presented in Table 5. No differences were noted in BW at any time during the 63-d receiving period (*p* ≥ 0.18). Average daily gain calculated on a deads-out basis was not different from D0–21, D0–43 nor from D0–63 (*p* ≥ 0.25). However, from D22–42, CON tended to have greater ADG compared with BXA (*p* = 0.11) but from D43–63 BXA had greater ADG compared with CON (*p* = 0.03). Likewise, G:F tended to be different (*p* ≥ 0.06), with more efficient gain observed on D22–42 and D0–42 for cattle fed CON. However, for D43–63 a tendency for greater feed efficiency was noted for BXA (*p* = 0.07). Average daily gain calculated on a deads-in basis followed similar trends, with no differences noted between treatments on D0–21, D22–42, D0–42 and D0–63 (*p* ≤ 0.77). Between D43 and D63, cattle assigned to the BXA treatment had greater ADG compared with CON (*p* = 0.03). Likewise, cattle fed BXA were more efficient from D43–63 (*p* = 0.05) and no differences were noted at any other time period. By design of the experiment, feed intake was limited to a percentage of pen BW (2.5%). However, target feed intakes were not achieved until D18 for most pens regardless of treatment and not until D38 for all pens. This is most likely due to health challenges that these cattle faced. Due to these discrepancies in achieving targeted intake, cattle fed BXA tended to have greater DMI from D0–21, D0–42 and from D22–42 (*p* ≤ 0.13). However, no differences in DMI were noted from D43–63 or from D0–63 (*p* ≥ 0.16), after targeted intakes were achieved.

**Figure 1:**
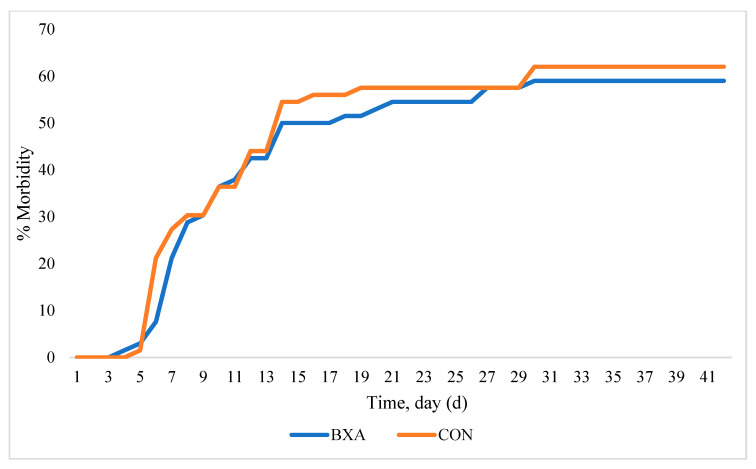
Cumulative morbidity across the 63 d receiving period.

As mentioned previously, health proved to be challenging, with overall morbidity for the BXA treatment group being 59.7% and 71.2% for the CON treatment group. However, morbidity was not different between treatments (*p* = 0.19). The percentage of mortality did not differ between treatments (*p* = 0.63) nor did the percentage of cattle completely removed from the study (chronically ill + dead). The distribution of morbidity across d is shown in Figure 1. There were no effects of treatment (*p* = 0.84), and no effects of treatment × day (*p* = 0.36). However, there was an effect of d (*p* < 0.001). Cost of morbidity (medicine cost + chute fee), not including metaphalaxis, tended to be less for BXA (*p* = 0.10).

### 3.2. Grazing Period

Data from the grazing period are presented in Table 6. No differences were noted at any timepoint for BW (*p* ≥ 0.29). Average daily gain for the grazing period was calculated based upon initiation of grazing. A tendency (*p* ≥ 0.06) was noted in ADG from D0–28, D29–56, D57–84, D0–112 and D0–126 with cattle supplemented with BXA having a tendency for greater gain than cattle supplemented with soybean hulls alone (CON). Moreover, the BXA treatment group had statistically greater ADG from D0–56 and from D0–84. No differences were observed from D84–112 (*p* = 0.82). It should be noted that from D103–111 abnormally heavy rains affected the area, which impacted how much supplement each pasture could consume, with several pastures not receiving any supplement during this period. This may have affected the performance of cattle during that period. Total ADG from D0 of the receiving period all the way through the termination of grazing was not different between treatments (*p* = 0.27).

## 4. Discussion

### 4.1. Receiving Period

Dry matter intake in high-risk beef cattle can be compromised due to the stresses of adaptation to a new environment as well as health challenges faced by these calves [2,23]. Despite these challenges it was observed in the present study that BXA tended to be greater for cattle fed BXA from D0–21, D22–42, and D0–42. In contrast, other studies have reported that similar products resulted in no effect on DMI. During the latter part of the study (D43–63 and D0–63) DMI did not differ. By design of the study, DMI should not have differed. However, it was observed that, during this study, cattle in both treatments did not fully reach their targeted intake until midway through the study. Feed increases were undertaken only when the pen had consumed all of the previous day’s deliveries for two consecutive d until they reached their targeted endpoint (2.5% of BW), therefore, despite not achieving their targeted intake, cattle fed BXA tended to have greater intake for 2/3 of the study. Lower levels of tannin supplementation have resulted in an increase in DMI of feedlot cattle. Similar to the present study Tabke et al. [24] determined that supplementing a similar tannin blend to that used in the present study resulted in improved DMI during the first half of the feeding period. Additionally, Bowman-Schnug et al. [25], found that feeding a similar blend resulted in improved DMI. However, it should be noted that other studies have shown that supplementing tannins resulted in a decrease in DMI [26]. Perhaps this increase in DMI was due to improved rumen function. In a meta-analysis, Berca et al. [19] determined that the use of tannins resulted in increased production of propionate and butyrate, which suggests improved fermentation. Perhaps the antioxidant properties of this tannin blend resulted in a reduction of that inflammation in these cattle.

Despite these tendencies, with DMI observed in the first part of the receiving period, no differences were noted on ADG from D0–21 on either a deads-out or deads-in calculation, and a tendency was observed for greater ADG (deads-out) for CON from D22–42. Despite the lack of difference in ADG for the first 2/3 of the study, ADG was greater for BXA from D43–63, in both deads-out and deads-in calculations. Data have noted positive responses in growth to the use of tannins and polyphenols, with the response being attributed to greater AA provided to the small intestine [15]. Similarly, blends of essential oils and tannins have resulted in positive effects on growth [17,20]. It is unclear why this difference between performances occurred within this receiving period. Based upon calculated energy values from proximate analysis (Table 3), it was noted that BXA had less Mcal/kg of net energy, which might explain these differences in ADG despite tendencies for greater DMI. As noted previously, DMI did not reach target intakes until approximately halfway through the receiving period. Rivera et al. [23] fed yeast products to newly received cattle and observed that cattle did not reach target intake of yeast products until 7 d following arrival. These researchers noted no differences in performance or health initially but tendencies for improved performance later in the study period. They hypothesized that the delay in achieving the target intake may have shifted the response back to later in the receiving period. Based upon the consistent responses noted with ADG between both methods of calculation from D43–63, we hypothesize that targeted levels of BXA were perhaps insufficient to elicit a growth response. Due to the high number of cattle that either died or were removed from the study, no differences were noted in feed efficiency (deads-in calculation). However, on a deads-out calculation, feed efficiency followed the same trend as ADG, with a significant positive response noted from D43–63.

Health was not impacted by treatment. Percentage of morbidity was similar between treatments. Data have suggested that the use of polyphenols from grape leaves might have a positive effect on animal health [12], with greater antibody production noted in vaccines. It should be noted that, in the study by Engler et al. [12], antibody titers were increased at D28 and D60. Although the cattle used by Felizari et al. [26] did not have the level of morbidity that cattle in the present study did, those authors noted no difference in respiratory morbidity either. While polyphenols might have effects on improving antibody titers, in newly received cattle this effect might be too late to impact health, as the majority of morbid cattle was reached by D21, and as a whole, cattle were not yet achieving targeted intakes. Despite the lack of response in percentage of morbidity, the overall medicine cost per animal tended to be lower with BXA compared with CON.

In general, no differences were noted for the first 2/3 of the receiving period, with the exception of tendencies for increased feed intake. Therefore, we hypothesize that, due to animals not achieving target intake goals, cattle were not consuming sufficient BXA to impact performance until the last 1/3 of the study, where treatment effects were noted.

### 4.2. Grazing Period

Supplementing grazing cattle with tannins and tannin-based additives after the receiving period can be beneficial for improving feed efficiency and weight gain as well as improving overall animal performance [27]. However, when cattle transition from the receiving period to a grazing system, the impacts of tannin supplementation become more complex as animal performance will be influenced by the type of tannin utilized, forage quality, as well as previous diet. In the current study, grazing data are more clearly defined, with no differences noted in BW at any time during the grazing period. However, ADG either tended to be increased or was statistically increased with the use of BXA for almost all periods of the study with the exception of D85–112. The results of the current study are like those of Min et al. [28] who found no differences in BW between ruminally cannulated steers grazing winter wheat (*Triticum aestivum* L.) that either received no tannin additive or 1 of 2 levels of quebracho-based tannin until D60. Additionally, ADG was greater for steers supplemented with a greater level of tannin-based additive as compared with steers on the control diet. However, while results are similar to those of the current study, in the study by Min et al. [28] prior diet/treatment of steers is unknown. Furthermore, only six steers were allocated per treatment (*n =* 18 total) indicating that results should be interpreted carefully in comparison to the results of this study.

Research suggests that finding an optimal CP to CT ratio is crucial to maximizing animal performance. If dietary protein is not limiting, tannins may enhance animal gains. In the aforementioned study by Min et al. [28], it was hypothesized that improved animal performance (ADG) in steers grazing winter wheat was in response to an optimum ratio of CP to CT. Although only one level of tannin blend supplementation was evaluated in the current study, it is possible that positive ADG responses were a result of an optimal CP:CT ratio within the diet as CP generally did not appear to be limiting during the grazing period. Additionally, no negative impacts related to tannin supplementation, such as decreased intake, were observed. Reviews evaluating the results of CT-containing diets fed to sheep have shown that positive animal responses were observed when CT were 2–3% DM within the diet [29,30]. Other studies have reported that CP:CT ratios were the most important factor on ADG both in grazing steers [31] and feedlot cattle [32]. In contrast to the positive impacts of CT, if CT is too high in the diet, fiber digestion can be negatively impacted, leading to reduced animal performance in cattle [32] and sheep [33]. However, these negative impacts could also be due to sensitivity of diet preferences [30] as well differences in rumen microbial diversity [34,35].

In the current study, we could hypothesize that, while BW did not differ between treatments, there may be differences in rumen microbial diversity that account for positive impacts on animal ADG. This is supported by the results of Min et al. [28]. As previously discussed, tannins generally act by binding to proteins, potentially altering the activity and composition of rumen microbes, including those responsible for CH_4_ production, such as methanogens [28]. By reducing rumen methanogenesis, energy efficiency of grazing cattle could be improved as CH_4_ represents a dietary energetic loss. This improved energy efficiency could contribute to better growth and increased ADG. Min et al. [28] indicated that CT improved ADG in steers grazing winter wheat in their in vivo experiment. This was supported by a reduction in CH_4_ with associated microbiota changes due to CT supplementation in their in vitro experiment. While we can speculate that improved animal performance observed in the grazing period was related to microbiological changes within the rumen environment, neither rumen microbial diversity nor CH_4_ were evaluated within this study, warranting further research related to the role of tannins and their impacts on grazing animal performance. Additionally, it must be realized that the ability of CT to bind proteins is not always directly related to the inhibition of CH_4_ production, indicating that the mechanism of action of tannins cannot solely be attributed to its ability to bind and inhibit methanogens [28,36]. This indicates that improved animal performance is based upon several factors, including but not limited to the type and dose of tannin used, duration of supplementation, and diet composition [37].

## 5. Conclusions

Feeding tannins during the receiving period can have positive impacts on animal performance during the subsequent grazing or feed period [38]. However, the effects can be variable and depend on several factors. Additionally, research related to tannin supplementation on the performance and health of high-risk beef cattle during the receiving period and subsequent grazing period is lacking. One objective of this study was to determine if tannin supplementation impacted health and performance of high-risk cattle during the receiving period. The main findings showed that supplemental tannin inclusion generally did not improve animal performance and health during the receiving period, potentially due to poor intake and health challenges faced by these cattle. Nonetheless, there were positive impacts on ADG during the subsequent grazing period. It could be hypothesized that positive impacts on ADG during the grazing period may be due to improved protein utilization and reduction in CH_4_, leading to enhanced energy efficiency. Nonetheless, without accurate measures of intake (which is difficult to do in grazing systems), it is unclear whether these differences in performance observed during grazing were due to greater efficiency or greater intake. Moreover, further research is required to fully understand the mechanism of action tannins have on ruminal microbial diversity, as neither CH_4_ nor metrics related to protein utilization were evaluated in the grazing portion of this study.

## Figures and Tables

**Table 1:** Ingredient composition (% DM) of the receiving diets fed to beef cattle over a 63-d receiving.

Ingredient	BXA ^1^	CON
Soybean hull pellets	26.33	29.66
Corn gluten feed pellets	25.00	25.00
Beet pulp pellets	15.00	15.00
Corn, DDGS	10.74	10.14
LNC Roughpel ^2^	10.00	10.00
Corn, cracked	6.75	5.51
BXA pellet ^3^	3.38	0.00
Liquid conditioner	2.50	2.50
Mineral pellet ^4^	0.00	1.50
Monensin pellet ^5^	0.30	0.30
Limestone	0.00	0.24
Salt	0.00	0.15

^1^ Cattle received one of two experimental diets: (1) a control diet (CON) or (2) a similar diet supplemented with the BXA pellet (BXA). ^2^ Roughage-based pellet consisting of low-quality hay and corn stalks. ^3^ Tannin blend pellet designed to deliver 7 g of BX and 1 g of ATX per head daily. ^4^ Mineral pellet providing trace mineral package. ^5^ Designed to provide 33 mg/kg of monensin.

**Table 2:** Clinical illness score used to initially determine morbidity of beef cattle during the 63-d receiving period [22].

Clinical Illness Score	Description	Appearance
1	Normal	No abnormal symptoms
2	Slightly ill	Mild depression, gaunt, may have nasal and ocular discharge
3	Moderately ill	Nasal and ocular discharge, lags behind cohorts, coughing, labored breathing, weight loss
4	Severely ill	Severe depression, labored breathing, purulent ocular/nasal discharge, not responsive to human approach
5	Moribund	Near death

**Table 3:** Chemical (% DM) composition of diets fed to beef cattle over a 63-d receiving period.

Item	BXA ^1^	CON
Crude protein (CP), %	15.5	15.2
Fat, %	1.70	1.50
Acid detergent fiber (ADF), %	24.3	25.2
Total digestible nutrients (TDN), %	72.8	75.5
Net energy maintenance (NE_m_), Mcal/kg	1.67	1.74
Net energy gain (NE_g_), Mcal/kg	1.10	1.19

^1^ Cattle received one of two experimental diets: (1) a control diet (CON) or (2) a similar diet supplemented with the BXA pellet (BXA).

**Table 4:** Chemical (% DM) composition of cool-season pastures grazed by beef cattle over a 126-d grazing period.

Period	CP, % DM	TDN, % DM
December–January	25.8	67.4
January–February	18.6	73.7
February–March	30.2	75.8
March–April	26.7	80.1
April (study termination)	22.1	76.8

**Table 5:** Animal performance and health of beef cattle receiving a tannin blend additive during the 63-d receiving period.

Item	BXA ^1^	CON	SE	*p*-Value
Body weight (BW), kg				
D0	180.6	180.1	0.8	0.63
D21	191.6	190.5	2.0	0.61
D42	211.8	213.6	2.4	0.51
D63	241.0	237.8	2.0	0.18
Dry matter intake (DMI), kg/d				
D0–21	3.11	2.91	0.11	0.12
D22–42	4.58	4.42	0.08	0.09
D0–42	3.78	3.61	0.09	0.13
D43–63	5.97	5.61	0.12	0.50
D0–63	4.31	4.17	0.09	0.16
Average daily gain (ADG), deads-out, kg/d ^2^				
D0–21	0.52	0.49	0.08	0.70
D22–42	0.98	1.10	0.07	0.11
D0–42	0.74	0.79	0.08	0.33
D43–63	1.38	1.15	0.08	0.03
D0–63	0.96	0.91	0.3	0.25
ADG, deads-in, kg/d ^3^				
D0–21	−0.63	−0.46	0.47	0.75
D22–42	−0.94	−0.86	0.79	0.91
D0–42	−0.75	−0.64	0.39	0.77
D43–63	1.23	0.95	0.10	0.03
D0–63	−0.20	−0.23	0.31	0.92
Gain:feed (G:F), deads-out, kg:kg				
D0–21	0.17	0.17	0.02	0.90
D22–42	0.21	0.25	0.02	0.07
D0–42	0.19	0.22	0.01	0.06
D43–63	0.24	0.21	0.02	0.07
D0–63	0.22	0.22	0.01	0.98
G:F, deads-in, kg:kg				
D0–21	−0.20	−0.22	0.17	0.93
D22–42	−0.22	−0.25	0.20	0.88
D0–42	−0.21	−0.23	0.15	0.90
D43–63	0.22	0.17	0.02	0.05
D0–63	−0.05	−0.09	0.10	0.70
Morbidity, %	59.7	71.2	5.99	0.19
Mortality, %	13.3	18.1	8.22	0.63
Chronic, %	16.6	10.8	5.09	0.90
Medicine cost/USD per head ^4^	12.95	15.56	1.38	0.10

^1^ Cattle received one of two experimental diets: (1) a control diet (CON) or (2) a similar diet supplemented with the BXA pellet (BXA). ^2^ Deads-in calculations left the dead animal in the total calculations. ^3^ Deads-out calculations removed the dead animal from all calculations. ^4^ Medicine costs for treatment of BRD, not including metaphalaxis, total costs divided by total head count at arrival.

**Table 6:** Performance of beef cattle receiving a tannin blend additive during the 126-d grazing period.

Item	BXA ^1^	CON	SE	*p*-Value
Body weight (BW), kg				
D0	238.2	239.2	8.4	0.95
D28	259.9	256.4	7.7	0.76
D56	290.3	281.3	7.9	0.53
D84	325.9	311.2	9.1	0.39
D112	362.0	347.5	9.1	0.29
D126	375.1	360.9	9.2	0.33
Average daily gain (ADG), kg/d				
D0–28	0.74	0.62	0.05	0.06
D29–56	1.06	0.89	0.10	0.15
D0–56	0.90	0.76	0.06	0.04
D57–84	1.23	1.08	0.08	0.09
D0–84	1.01	0.87	0.06	0.05
D85–112	1.29	1.27	0.06	0.82
D0–112	1.08	0.97	0.06	0.08
D0–126	1.07	0.97	0.05	0.09
Total (receiving + grazing) ADG, kg/d ^2^	0.49	0.38	0.09	0.28

^1^ Cattle received one of two experimental diets: (1) a control diet (CON) or (2) a similar diet supplemented with the BXA pellet (BXA). ^2^ Total ADG was calculated from D0 of the 63-d receiving period through the termination of the 126-d grazing period.

## Data Availability

Data that supports the findings of this study are available from the authors upon request.

## Abbreviations

The following abbreviations are used in this manuscript:

BXA	Diet supplemented with the BXA pellet (7 g BX and 1 g ATX per head daily)
CON	Control diet
ADG	Average daily gain
BRD	Bovine respiratory disease
CH_4_	Methane
BW	Body weight
DM	Dry matter
PTI	Post-treatment interval
RT	Rectal temperature
DMI	Dry matter intake
G:F	Gain:feed
CP	Crude protein
ADF	Acid detergent fiber
TDN	Total digestible nutrients
NE_m_	Net energy of maintenance
NE_g_	Net energy of gain
CT	Condensed tannins
HT	Hydrolysable tannins
